# Fever: A Clinical Study

**Published:** 1889-03

**Authors:** 


*Fever: a Clinical Study.* By T. J. MACLAGAN, M.D. London: J. & A. CHURCHILL. 1888.

Dr. Maclagan has above all things the pen of a ready writer, and this book forms no exception in respect of quality to his previous publications. It is so pleasant to peruse a book whose grammar is irreproachable, and style attractive. In the space of something over 160 pages, Dr. Maclagan enunciates his theories and views upon fever, its causes, phenomena, and treatment; gliding with easy confidence over the choppy seas—so to speak—caused by the conflicting currents of knowledge, half-knowledge, and ignorance. He gives with moderate fulness the views of others on fever, such as Virchow, H. C. Wood, Miller Ord, Hale White, and, still more recently, Macalister; in order, however, to dissent from them, more or less. He quotes with approbation only Jenner, writing more than thirty years ago. Without attempting to give Dr. Maclagan's own views on fever, an epitomised idea of his position may be gathered from these brief quotations:—

"There is really no valid reason for supposing that during fever the mutual relation of heat-production and heat-elimination is materially altered."

"In all fevers the rise of temperature results from increased metamorphosis."

"Hyperpyrexia may be defined as paralysis of inhibition of metabolism; paralysis of heat-inhibition being only part of the process."

"There is not sufficient evidence to show that this special function (influence over heat-production) is localised in any one part of the nervous centres."

These sentences indicate a considerable divergence of opinion from the views of Dr. Macalister, or those expressed at the discussion on fever, at the American Physiological Society some months ago, which are those now most usually held.

Dr. Maclagan prefers the study of fever from the clinical side to that from the physiological or pharmacological side; but not much more can we hope to gain, now-a-days, from the clinical investigation of such a subject as fever, upon which purely clinical medicine has practically exhausted its resources. Physicians must look to the physiologists to further unravel its unexplained mysteries. In another edition we trust that the important researches of Sawdoski and Van Noorden into the action of antipyretics will be fairly considered, and the bearings of those investigations incorporated in the text.

